# Identification and Evaluation of Traditional Chinese Medicine Natural Compounds as Potential Myostatin Inhibitors: An In Silico Approach

**DOI:** 10.3390/molecules27134303

**Published:** 2022-07-04

**Authors:** Shahid Ali, Khurshid Ahmad, Sibhghatulla Shaikh, Jeong Ho Lim, Hee Jin Chun, Syed Sayeed Ahmad, Eun Ju Lee, Inho Choi

**Affiliations:** 1 Department of Medical Biotechnology, Yeungnam University, Gyeongsan 38541, Korea; ali.ali.md111@gmail.com (S.A.); ahmadkhursheed2008@gmail.com (K.A.); sibhghat.88@gmail.com (S.S.); lim2249@naver.com (J.H.L.); po98053@gmail.com (H.J.C.); sayeedahmad4@gmail.com (S.S.A.); gorapadoc0315@hanmail.net (E.J.L.); 2 Research Institute of Cell Culture, Yeungnam University, Gyeongsan 38541, Korea

**Keywords:** myostatin, myogenesis, natural compounds, MD simulations, muscle-related diseases, traditional Chinese medicine

## Abstract

Myostatin (MSTN), a negative regulator of muscle mass, is reported to be increased in conditions linked with muscle atrophy, sarcopenia, and other muscle-related diseases. Most pharmacologic approaches that treat muscle disorders are ineffective, emphasizing the emergence of MSTN inhibition. In this study, we used computational screening to uncover natural small bioactive inhibitors from the Traditional Chinese Medicine database (~38,000 compounds) for the MSTN protein. Potential ligands were screened, based on binding affinity (150), physicochemical (53) and ADMET properties (17). We found two hits (ZINC85592908 and ZINC85511481) with high binding affinity and specificity, and their binding patterns with MSTN protein. In addition, molecular dynamic simulations were run on each complex to better understand the interaction mechanism of MSTN with the control (curcumin) and the hit compounds (ZINC85592908 and ZINC85511481). We determined that the hits bind to the active pocket site (Helix region) and trigger conformational changes in the MSTN protein. Since the stability of the ZINC85592908 compound was greater than the MSTN control, we believe that ZINC85592908 has therapeutic potential against the MSTN protein and may hinder downstream singling by inhibiting the MSTN protein and increasing myogenesis in the skeletal muscle tissues.

## 1. Introduction

Skeletal muscle (SM) is the largest tissue mass in the body, accounting for 40–45% of total body mass [1]. SM helps perform essential functions of the body such as movement, body support, temperature control, and balancing glucose levels. In addition, SM has the ability to regenerate in response to injury or disease with the assistance of muscle satellite cells (MSCs), which can self-renew and generate differentiated progeny [2]. Coordination of the expression of paired box transcription factors (Pax3/Pax7) and the basic helix-loop-helix family of transcription factors, which includes myogenic factor 5, myogenic differentiation, and myogenin, is required for the proliferation and differentiation of MSCs to form myotubes via myogenesis [3]. The balance of protein production and degradation is critical for the maintenance of SM, and is extremely sensitive to hormonal balance, exercise, injury, malnutrition, and disease [4].

MSTN is a member of the transforming growth factor (TGF-β) family that has been documented as a potent muscle growth inhibitor [5]. Clinical studies have explored the connection between MSTN and muscle-wasting diseases [6]. Muscle mass in MSTN knockout mice is 2–3 times more frequent than in wild type mice [7]. Furthermore, the transgenic overexpression of MSTN inhibitors such as follistatin (Fst) or the dominant-negative version of the receptor ActRIIB results in a similar phenotype [8]. Extracellular matrix proteins such as fibromodulin (FMOD), decorin, fibronectin (FN), and laminins bind to MSTN and modulate its function [9]. We previously investigated a number of ECM proteins that are involved in the regulation of myogenesis, including FMOD [10,11], matrix Gla protein [12], and dermatopontin [13]. FMOD slows muscle aging by suppressing the MSTN gene or decreasing MSTN protein activity, whereas MSTN promotes muscle aging [14]. Co-immunoprecipitation studies revealed that FMOD interacts directly with MSTN during myoblast differentiation. Moreover, protein–protein interaction between FMOD and MSTN and its receptor (ACVRIIB) showed that FMOD effectively reduces the MSTN-ACVRIIB interaction [10].

MSTN has been the subject of intense research since its discovery, and MSTN inhibitors are now being investigated as prospective therapeutics for muscle-wasting illnesses such as muscular dystrophy and sarcopenia [15]. Recently, we established via an in silico analysis that natural compounds (curcumin and gingerol) suppress MSTN-ACVRIIB interaction [16]. To extend our search for novel MSTN inhibitors, we employed virtual high-throughput screening (vHTS) on Traditional Chinese Medicine (TCM) compounds. TCM has received growing interest in the life science sector as a traditional medical intervention in Asia and as a supplement and alternative therapy in Western nations. TCM offers a wealth of natural resources for medicinal compounds, and these resources are widely regarded as both useful and safe in the drug development. In the search for novel inhibitors that precisely target MSTN, we conducted structure-based vHTS on approximately 38,000 TCM compounds, followed by all-atom MD simulations to identify potent MSTN inhibitors.

## 2. Method and Materials

The experiments were conducted on the HPC server with an Intel^®^ Xeon^®^ Silver 4216 CPU running at 2.10 GHz which has 32 logical cores and 3 TB of data storage. This study made use of a number of computational tools, such as the following: PyRx 0.8 was utilized with the AutoDock Vina [17] for vHTS and molecular docking investigations; and PyMOL [18], VMD (visual molecular dynamics) [19], and Discovery studio visualizer [20] were used for visualization purpose.

### 2.1. Preparation of Target Protein and Natural Compounds Library

The PDB database was used to obtain the 3D structure of the MSTN protein (PDB ID: 3HH2). For further analysis, the structure was visually inspected and thoroughly cleaned using the Discovery studio 2021 software. A non-commercial ZINC database was used to create a library of natural compounds based on the TCM database, which contained about 38,000 small compounds [21]. The library contains 3D file formats of processed chemical structures of TCM natural compounds.

### 2.2. Evaluation of Potential Leads and Drug-Ability

Based on binding affinity (BA) and scoring, the top 150 hits with the highest BAs to the MSTN protein were chosen. In order to discover safe and effective drug-like molecules, the selected compounds were further examined for their physicochemical and ADME properties. Web-based software tools such as Swiss-ADME [22], PreADMET, and CarcinoPred-EL [23] were used to predict these properties, which included toxicity and carcinogenicity. The compounds were then examined for PAINS (Pan-assay interference compounds)-pattern using the SwissADME web server (http://www.swissadme.ch/) (accessed on 5 October 2021 [24] and ZINC15 chemistry pattern database (http://zinc15.docking.org/patterns/subsets/pains) (accessed on 7 October 2021 [25]. A further interaction analysis was performed to avoid false positives and to obtain selective compounds with high specificity towards the binding active pocket of the MSTN protein.

### 2.3. Visualization and Assessment of MSTN Protein

A visual assessment of docked conformations of compounds with the MSTN protein was performed using the visualization tools PyMOL, Discovery Studio (2021), and LigPlot+. These programs generate high-quality animated 3D and 2D figures of the MSTN protein and chemical compounds. Aside from visual inspections, various parameters were determined, including bond length, distance between residues, and distance between the MSTN protein and compounds.

### 2.4. Molecular Dynamics (MD) Simulations

MD simulations of the MSTN-Curcumin, MSTN-ZINC85592908, and MSTN-ZINC85511481 complexes were performed at 300 K using the GROMACS 2019.6 [26]; the GROMOS96 43a1 force-field was subsequently obtained [27]. The PRODRG server was used to generate the compound topology and force-field parameters [28]. The atoms of the three compounds (Curcumin, ZINC85592908 and ZINC85511481) were combined in complex topology files. The charges on the MSTN protein complexes were neutralized by introducing Na+ and Cl- ions using the gmx_genion module (0.15 M). The particle-mesh Ewald method [28] was used to investigate the interactions of MSTN with the compounds, using energy-grps in the MD parameters (mdp) file. The MD system was then minimized using the steepest descent (1500 steps). The temperature was subsequently raised (0 to 300 K) over a 100-ps equilibration period under periodic boundary conditions at a constant volume.

The equilibration process was completed in the following two stages: NVT and NPT ensembles. Following that, the final production phase (100 ns) was achieved at 300 K. The resulting trajectories were investigated using the GROMACS analysis modules. The graphical presentations of the 3D models were prepared using VMD [19] and PyMOL.

## 3. Result and Discussion

Increased MSTN protein expression is commonly linked to muscular atrophy, which is frequently encountered in cancer, HIV infection, burn injury, aging, muscle incapacity, sarcopenia, and sepsis [29,30,31]. MSTN is a myocyte-secreted protein that acts as a negative regulator of SM mass and growth [32]. In the current study, we screened about 38,000 small compounds from the TCM Database against MSTN. The top 150 compounds with the highest BAs were subjected to further analysis. Using several tools, all new hits were precisely identified within the MSTN pocket and analyzed for drug-likeness.

### 3.1. Active Pocket Analysis

The natural inhibitor of MSTN is follistatin (fst), which binds to MSTN and forms a complex structure.

**Figure 1:**
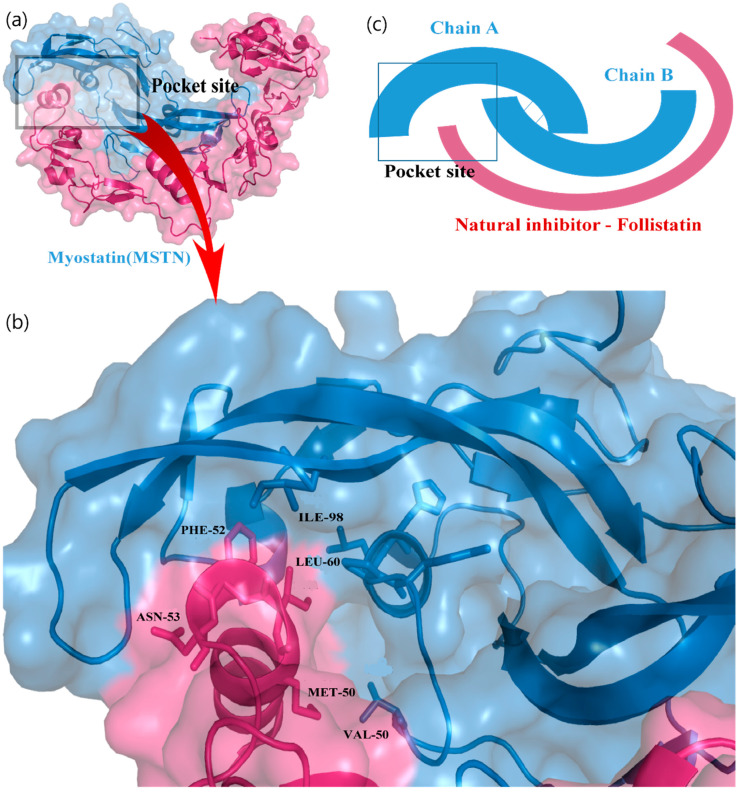
Identification of active site of MSTN protein. (**a**) MSTN-follistatin288 complex structure, (**b**) enlarge view of helix-helix interaction with MSTN. (**c**) Cartoon model of active pocket of MSTN protein.

Fst288 binds with MSTN via a helix–helix interaction (Figure 1a,b) near the C-terminal of the MSTN protein [33]. The MSTN C-terminal provides a groove-like structure to bind fst288 in the cavity, forming a closed packing. Finally, the fst288 hinders further signaling via the ActRIIB receptor. The most important residues of fst288 are Ile51, Met50, Phe52, and Asn53, and the residues of MSTN are Leu60, Pro56, and His59. Most interactions were determined to be within the helix residues. Therefore, the MSTN C-terminal site was very important for targeting the design and screening of drugs. We predicted a specific binding pocket of MSTN-fst288 complex that shows the active site to screen natural compounds (Figure 1c).

### 3.2. Molecular Docking, Hit Selection, and Drug-Ability Assessment

The screening of the TCM compound library resulted in log and output files including BA scores and docked postures for each compound in the library. These log and output files were analyzed for BAs and binding orientation for the MSTN protein. In the search for potentially active MSTN protein inhibitors, several natural compounds with a high BA score were chosen. The hits were filtered to obtain compounds with the highest BA from the 38,000 screened-compounds, which gave us 150 natural compounds (Table S1). These compounds were then filtered based on their physicochemical properties, with 53 compounds qualifying with specific drug-likeliness cut-off values. The compounds were chosen based on criteria such as H-bond donors ≤5, H-bond acceptors ≤10, rotatable bonds ≤10, molecular weight ≤750 Dalton, and logP ≤10 (Table 1).

However, some compounds, such as several FDA-approved drugs, breached the Lipinski rule of five because their molecular weight was greater than 500 Dalton and logP value was greater than five; however, this breach was deemed acceptable [34]. The ADMET properties were anticipated for these compounds, and 17 were found to be acceptable (Table 2). All these findings determined that the small natural compounds exhibit optimal drug-like molecular behaviors.

In addition, interaction analysis was used to find hits unique to the MSTN protein pocket site, and the two best compounds (ZINC85511481 and ZINC85592908) were subsequently selected, along with one compound chosen as a reference inhibitor (Curcumin) [16]. The two hits were passed through the Pan-assay interference compounds (PAINS) filters, and no PAINS pattern alerts were discovered, indicating that the compounds are MSTN specific. Based on these findings, we postulated that ZINC85511481 and ZINC85592908 are possible MSTN inhibitors with high BA and specificity for the MSTN binding pocket, and work by decreasing the MSTN accessibility to the ActRIIB receptor complex.

### 3.3. Interaction Analysis of MSTN Complexes

**Figure 2:**
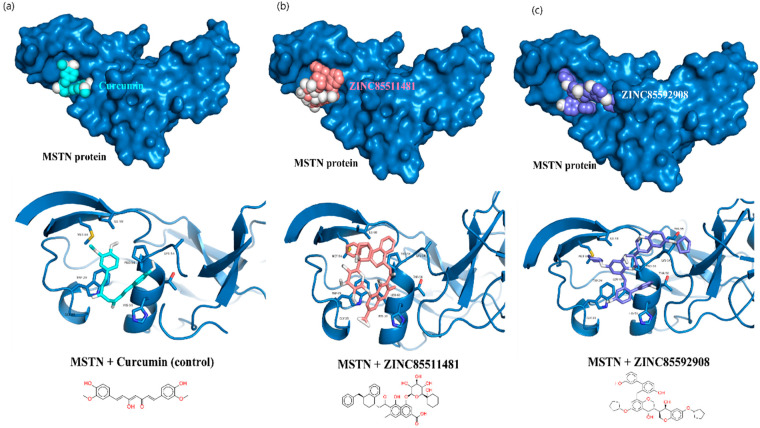
Interaction analysis of potential leads in their active pocket site, (**a**) MSTN-Curcumin, (**b**) MSTN-ZINC85511481 and (**c**) MSTN-ZINC85592908.

The structural analysis of MSTN complexes suggested that the catalytic pocket consists of an alpha helix element, and that helix residues (His59, Pro56 and Val50) are involved in the interaction with fst288. The interaction of the final leads was analyzed with the MSTN protein. Several interactions were determined within the active pocket of the MSTN–Curcumin complex. Curcumin demonstrated two van der Waals interactions with the MSTN protein residues Val50 and Pro56, as well as a sigma interaction with Val50 (Figure 2a). The MSTN-ZINC85511481 complex formed two hydrogen bonds with Gln53 and Pro56, an unfavorable interaction with the His59 residue, as well as a π-alkyl bond with Pro56 (Figure 2b). However, curcumin shows better interaction with MSTN than ZINC85511481.

The MSTN-ZINC85592908 complex interacted with Gln53 and Pro56 via two hydrogen bonds. It also formed three π-alkyl interactions with the Lys54 and Pro56 residues. ZINC85592908 showed a more favorable interaction with the MSTN protein at the active site, as compared to Curcumin and ZINC85511481. Furthermore, the stability of these complexes was examined through molecular dynamic simulation studies.

### 3.4. Molecular Dynamics Trajectory Analysis of MSTN-Ligand Complexes

The stability profiles of Curcumin, ZINC85592908, and ZINC85511481 in the complex with the growth and differentiation factor-8 (MSTN) were examined using the GROMACS module gmx_rmsd to assess their respective RMSD values throughout the simulation runs. In general, RMSD is a vital fundamental parameter for identifying whether a protein is stable and adheres to its experimental structure [21]. Thus, high RMSD values, associated with instability, indicate changes in the conformation of a protein. In MD simulations of protein–ligand, the RMSD average values for MSTN–Curcumin, MSTN–ZINC85592908, and MSTN–ZINC85511481 were 0.49, 0.50, and 0.55 nm, respectively. The RMSD plot showed that the MSTN–Curcumin and MSTN–ZINC85592908 binding imparted better stabilization to the MSTN protein and resulted in lesser structural deviations from its normal conformation. In contrast, the MSTN–ZINC85511481 complex showed high deviation with the MSTN protein.

**Figure 3:**
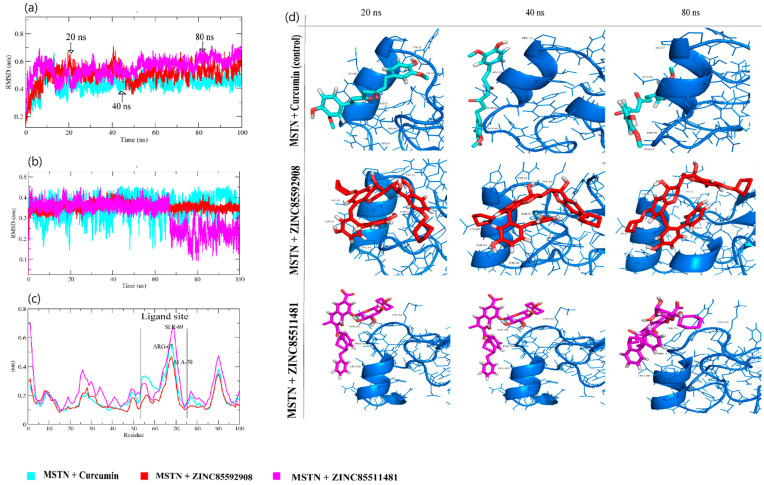
Conformational studies of complexes. (**a**) Root mean square deviation of backbone of MSTN protein, (**b**) Root mean square deviation of ligands, (**c**) Root mean square fluctuation of MSTN protein, (**d**) snapshots of complexes with different interval of time 20, 40 and 80 ns.

The results suggest that ZINC85511481 was unstable in the vicinity of the MSTN protein (Figure 3a). Moreover, snapshots from the MD trajectory of Curcumin and ZINC85592908 showed that these compounds interact with the helix region of the MSTN protein, whereas ZINC85511481 loses this interaction at the time of MD trajectory. At 80 ns, both the control and ZINC85592908 were shown to be stable and to interact better than ZINC85511481. (Figure 3d). Further analysis of the compound RMSD to determine the dynamic motion revealed that Curcumin and ZINC85592908 bind better than ZINC85511481 and are more stable (Figure 3b).

To gain more insights regarding the stability of the complex pocket site, the per residue root-mean-square fluctuation (RMSF) contour was determined for each ligand-bound protein. The exclusive backbone RMSF of each protein complex was estimated, and this RMSF provides detailed information about the contribution of individual protein residues within the ligand/protein complex structural fluctuations. MSTN–Curcumin, MSTN–ZINC85592908, and MSTN–ZINC85511481 backbones showed continuous fluctuations in the MSTN pocket site. Most likely the result of various orientations, with a high fluctuation region observed between residues 51–75 (Figure 3c), which is the helix region of the MSTN protein and, specifically ARG-67, SER-69 and ALA-70 residues. For the binding of ligands, the RMSF of MSTN was exhibited as a function of residue numbers to the MSTN protein, as well as the average fluctuation of all residues during the simulation. Moreover, the plot indicates that MSTN had residual variations in multiple areas, and the MSTN–Curcumin and MSTN–ZINC85592908 complexes minimized the residual fluctuations. The highest fluctuations were determined in the MSTN–ZINC85511481 complex. Taken together, these results indicate that the ZINC85592908 compound might be a better potential drug than ZINC85511481.

**Figure 4:**
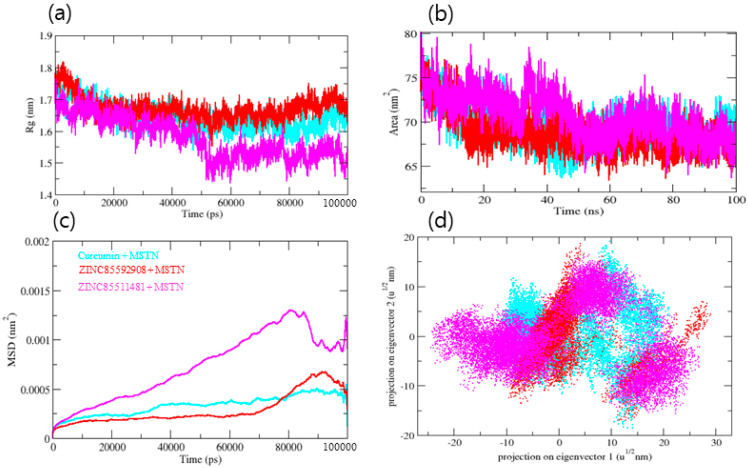
(**a**) Radius of gyration, (**b**) SASA of MSTN protein, (**c**) MSD (Mean square displacement) of MSTN protein, (**d**) Two dimensional projection of MSTN protein.

To gain insight into the complex stability/compactness profile in a biological system, we applied the Radius of gyration (Rg). The MSTN–Curcumin, MSTN–ZINC85592908, and MSTN–ZINC85511481 complexes had average Rg values 1.63, 1.66, and 1.54 nm, respectively. Stable Rg trajectories were observed for MSTN–Curcumin and MSTN–ZINC85592908 with decreased maximum, average, and lowest values, indicating compactness and stability of the ligand within the MSTN protein active pocket site. MSTN–ZINC85511481 also had comparable values (Figure 4a). We further investigated the Solvent Accessible Surface Area (SASA), which refers to the region of a protein’s surface that interacts with its solvent molecules [22]. Average SASA values for MSTN–Curcumin, MSTN–ZINC85592908, and MSTN–ZINC85511481 complexes were observed throughout the MD trajectory, with average SASA values of 69.66, 68.81, and 70.73 nm^2^, respectively (Figure 4b). SASA analysis showed that the ZINC85511481 compound was exposed more to the solvent, as compared to Curcumin and ZINC85592908. These results suggest that Curcumin and ZINC85592908 bind strongly to the MSTN protein and have less interaction with water molecules. Furthermore, the mean square displacement (MSD) of atoms from a collection of original MSTN protein complex positions was calculated (Figure 4c). The displacement of atoms from a set of initial positions in the MSTN–Curcumin, MSTN-ZINC85592908, and MSTN–ZINC85511481 complexes was estimated, with MSTN–ZINC85511481 exhibiting the highest MSD value.

Additionally, we also determined secondary structural assignments in MSTN proteins such as -helix, -sheet, and turn which were fragmented into specific residues during the simulations. Because of the enhanced fraction of coils and decrease in -sheet, the average number of residues involved in secondary structure formation in complexes was lowered. The MSTN protein in the MSTN–Curcumin and MSTN–ZINC85592908 complexes showed similar compositions of secondary structure element during the simulation (Table S2). In the case of MSTN–ZINC85511481, the proportion of strand element was observed to be considerably lower and the composition was altered upon the binding of ZINC85511481 to the MSTN protein.

**Figure 5:**
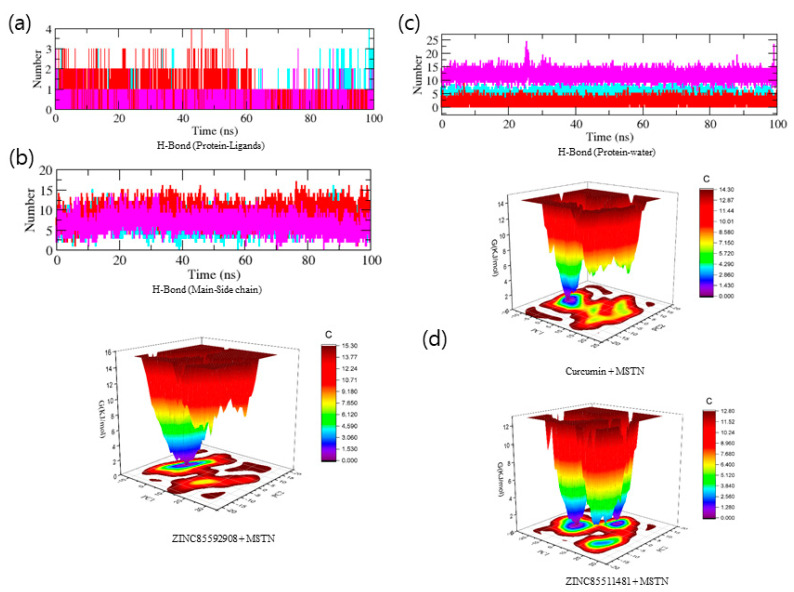
(**a**–**c**) Hydrogen bonding analysis between the MSTN protein and natural compounds, (**d**) GFE landscape plot for complexes.

To recognize the binding interaction pattern of compounds with the MSTN protein, we performed a hydrogen bond analysis. The H-bond is vital for the stability of the ligand-protein complex [25]. The hydrogen bonds were observed to be paired within 0.35 nm between the protein and ligand. The MSTN-Curcumin and MSTN-ZINC85592908 complexes strongly bind to the MSTN pocket with 2–4 hydrogen bonds, whereas MSTN-ZINC85511481 binds to the MSTN pocket with 1–2 hydrogen bonds. Due to the low number of H-bonds, this complex shows more fluctuations in the pocket and forms lesser stable complexes with the MSTN protein. The H-bond analysis performed with protein and water revealed that the number of H-bonds was significantly higher in MSTN–Curcumin and MSTN-–INC85592908 complexes, as compared to MSTN–ZINC85511481 (Figure 5a–c). Moreover, the Gibbs’ free energy (GFE) landscape was also computed with GROMACS analysis modules and projections of their respective first (PC1) and second (PC2) eigenvectors. The Comparable GFE contour map with darker blue shades represents less energy. The global minima of MSTN fluctuated during the simulations due to the complexes binding to MSTN. MSTN–Curcumin and MSTN–ZINC85592908 showed similar projections, and MSTN–ZINC85511481 showed a different global minima, indicating that the ZINC85511481 global minima was drastically altered during the simulation (Figure 5d). Taken together, these results suggest that ZINC85592908 has the potential to be applied as a drug for MSTN protein inhibition and increase myogenesis in skeletal muscle tissues.

## 4. Conclusions

Identifying possible and specific MSTN protein inhibitors is a viable approach for treating muscle disorders such as aging, muscular incapacity, sarcopenia, and sepsis. In the current study, the TCM database was screened against MSTN to uncover promising and highly potent inhibitors. ZINC85592908 and ZINC85511481 were chosen among the TCM-screened compounds because of their high binding affinities and interactions with the active pocket site (helix region). These two best hits were identified based on multiple screenings and are expected to be potential MSTN inhibitors. Both compounds were subjected to several tests, including drug-likeness, ADME, and toxicity. All compounds chosen were found to interact with helix residues and occupy the same binding pocket as Curcumin (control inhibitor). The MD simulation study also revealed that these hits formed stable conformations with MSTN. Among all the identified potential inhibitors, ZINC85592908 was found to have the best active pocket site stability. Thus, we propose that ZINC85592908 is potentially a novel inhibitor that could pave the way for the development of more promising drugs for muscle-related disorders.

## Figures and Tables

**Table 1:** Physicochemical properties of the selected compounds.

S. No	Molecule Name	rBonds	MW (D)	LogP	LogS	H-Acceptors	H-Donors	Druglikeness	DrugScore
1.	ZINC85542646	6	743.105	9.8211	−11.256	4	4	2.1393	0.0482794
2.	ZINC85569060	14	742.93	7.2932	−9.232	8	7	1.1649	0.02769731
3.	ZINC85542795	8	735.126	9.3453	−9.439	4	4	3.5148	0.1333178
4.	ZINC85625736	13	732.871	8.4908	−9.179	10	6	−3.2413	0.025806
5.	ZINC85542639	6	715.051	9.1759	−10.588	4	4	2.7851	0.05082912
6.	ZINC85542627	6	717.067	9.5135	−10.833	4	4	2.1393	0.04927217
7.	ZINC85531289	6	720.856	8.5678	−8.717	9	1	−5.2949	0.02028264
8.	ZINC85542877	10	723.115	9.6422	−9.433	4	4	1.6451	0.1248953
9.	ZINC85542671	6	720.071	7.3836	−8.911	5	5	4.507	0.1466092
10.	ZINC85569094	14	720.924	7.7686	−8.987	8	7	−0.80112	0.03387373
11.	ZINC85532197	3	743.063	8.2324	−9.457	6	2	1.6002	0.1263831
12.	ZINC85532197_01	3	742.055	8.2324	−9.457	6	1	1.6002	0.1264543
13.	ZINC85511481	14	710.817	6.4144	−8.805	10	6	−3.0206	0.05147698
14.	ZINC85569082	14	692.87	7.0778	−8.648	8	7	−0.63016	0.03765389
15.	ZINC85542801	6	699.093	8.6257	−9.107	4	4	1.8161	0.1313846
16.	ZINC85542734	7	693.045	8.4862	−9.062	4	4	2.1393	0.1351283
17.	ZINC85596043	8	686.87	8.4597	−9.448	8	4	−5.2779	0.0430603
18.	ZINC85531399	6	678.819	8.8366	−7.533	8	1	−4.778	0.02747577
19.	ZINC85542810	6	685.066	8.2215	−8.82	4	4	1.5833	0.1331636
20.	ZINC85592913	2	678.819	7.9773	−8.865	8	4	−4.767	0.07450064
21.	ZINC85531346	3	676.803	7.8245	−7.602	8	1	−7.5213	0.02813334
22.	ZINC85542876	6	673.055	8.3219	−8.832	4	4	1.8161	0.1365408
23.	ZINC95911591	1	656.816	9.9572	−11.412	6	1	−3.9286	0.0727493
24.	ZINC85542793	6	671.039	8.0144	−8.677	4	4	1.5283	0.1360255
25.	ZINC85542803	6	671.039	7.9522	−8.66	4	4	1.3666	0.134584
26.	ZINC85592908	12	664.792	7.6353	−8.595	8	4	−2.4411	0.08293768
27.	ZINC85531409	5	650.765	7.8019	−6.708	8	1	−5.4649	0.03143037
28.	ZINC85530919	7	644.718	8.5529	−7.639	8	4	−1.1148	0.0982419
29.	ZINC85542903	6	645.001	7.7106	−8.402	4	4	1.5283	0.1435007
30.	ZINC85542935	6	645.001	7.6484	−8.385	4	4	1.3666	0.1421086
31.	ZINC85542917	6	645.001	7.6484	−8.385	4	4	1.5283	0.14408
32.	ZINC85592903	10	636.739	7.0594	−7.91	8	4	−5.2216	0.08549763
33.	ZINC85542926	5	616.947	7.2631	−7.795	4	4	3.5148	0.1706919
34.	ZINC85531359	7	620.693	5.5821	−5.48	10	2	−9.9673	0.04023146
35.	ZINC85543487	1	629.007	9.673	−9.675	2	2	−0.43731	0.1041854
36.	ZINC85949541	2	592.69	6.3122	−6.34	8	0	1.9873	0.1715774
37.	ZINC70454202_01	4	593.742	6.3699	−6.12	7	3	4.6261	0.2320921
38.	ZINC70454202	4	594.749	6.3699	−6.12	7	4	4.6261	0.2316766
39.	ZINC85543478	1	616.996	9.3076	−9.498	2	2	−0.52971	0.1050597
40.	ZINC85541065	3	576.691	7.2035	−8.002	7	1	4.6261	0.147236
41.	ZINC85531053	3	576.647	6.7914	−10.044	8	1	1.741	0.1367792
42.	ZINC42802834	2	562.664	7.0099	−8.195	7	1	4.6261	0.1527083
43.	ZINC85541288	2	562.664	7.0327	−8.871	7	1	4.8552	0.1497575
44.	ZINC95910145	2	548.637	6.757	−8.557	7	2	4.8369	0.1594868
45.	ZINC44086846	2	546.621	6.8386	−9.35	7	1	4.9691	0.1565135
46.	ZINC85991498_01	1	548.593	5.5895	−6.867	8	2	−1.7726	0.1196459
47.	ZINC85991498	2	548.593	5.5895	−6.867	8	2	−1.7726	0.1196459
48.	ZINC03780340	6	504.449	5.9594	−10.586	8	6	−1.1275	0.07115073
49.	ZINC14680812	6	512.513	2.823	−5.007	8	7	0.51052	0.2703446
50.	ZINC85596478	3	482.618	7.2172	−7.679	4	1	−6.4955	0.04087312
51.	ZINC85947357_01	3	525.814	8.162	−9.5	4	4	−2.6575	0.04786098
52.	ZINC04098631	3	440.494	6.1569	−7.512	5	3	−3.1957	0.1446293
53.	Curcumin	8	368.384	2.039	−3.622	6	2	−4.7745	0.391063

MW: Molecular weight (Dalton), LogP: Lipophilicity and rBonds: Rotatable bonds (measure of molecular flexibility of a compound).

**Table 2:** ADMET properties of the selected compounds for MSTN protein.

S. No.	Molecule	BBB Permeant	PAINS	WLOGP	TPSA	Log S	Skin Permeability	CYP2D6 Inhibitor	Carcinogenicity
1.	ZINC85542795	No	0	8.79	72.72	−12.48	−2.98	No	None
2.	ZINC85531289	No	0	7.4	121.5	−10.37	−5.04	No	None
3.	ZINC85542877	No	0	8.63	72.72	−12.42	−2.95	No	None
4.	ZINC85542671	No	0	7.73	84.75	−10.87	−4.17	No	None
5.	ZINC85541288	No	0	5.5	61.42	−6.92	−5.57	No	None
6.	ZINC85532197	No	0	8.31	127.62	−10.4	−5.24	No	None
7.	ZINC85511481	No	0	5.91	173.98	−9.86	−6.08	No	None
8.	ZINC85592908	No	0	7.13	117.84	−9.54	−5.21	No	None
9.	ZINC14680812	No	0	3.69	158.68	−4.19	−8.53	No	None
10.	ZINC85592903	No	0	6.14	117.84	−8.63	−5.67	No	None
11.	ZINC85531359	No	0	4.29	141.73	−6.83	−7.14	No	None
12.	ZINC70454202	No	0	4.59	92.21	−8.13	−5.38	No	None
13.	ZINC95910145	No	0	5.2	72.42	−6.82	−5.71	No	None
14.	ZINC44086846	No	0	5.68	72.75	−6.74	−5.76	No	None
15.	ZINC04098631	No	0	6.27	79.15	−7.88	−4.43	No	None
16.	ZINC85991498	No	0	5.39	109.94	−6.7	−6.34	No	None
17.	Curcumin	No	0	3.15	93.06	−4.83	−6.28	No	None

BB (Blood–brain barrier) penetration ability, GIA (Gastrointestinal absorption), PSA (Polar surface area): ≤90Å2 is the optimum value, LogS: water solubility.

## Data Availability

Not applicable.

## Supplementary Materials

The following are available online at https://www.mdpi.com/article/10.3390/molecules27134303/s1, Table S1: List of top 150 compounds showing apparent binding affinity; Table S2. Percentage of secondary structure element in MSTN protein.
