# Supplementary material for: Identification and Evaluation of Traditional Chinese Medicine Natural Compounds as Potential Myostatin Inhibitors: An In Silico Approach
Source: Molecules. 2022 Jul 4;27(13):4303. doi: 10.3390/molecules27134303 (PMC9268423; doi:10.3390/molecules27134303)
Supplement: Supplementary file 1 [file molecules-27-04303-s001.zip › molecules-1795206-supplementary.pdf]

(Supplementary data)

Identification and evaluation of traditional Chinese medicine natural compounds as potential myostatin inhibitors: An Insilico approach

Shahid Ali[1,2], Khurshid Ahmad[1,2], Sibhghatulla Shaikh[1,2], Jeong Ho Lim[1,2], Hee Jin Chun[1,2], Syed Sayeed Ahmad[1,2], Eun Ju Lee[1,2], Inho Choi[1,2*]

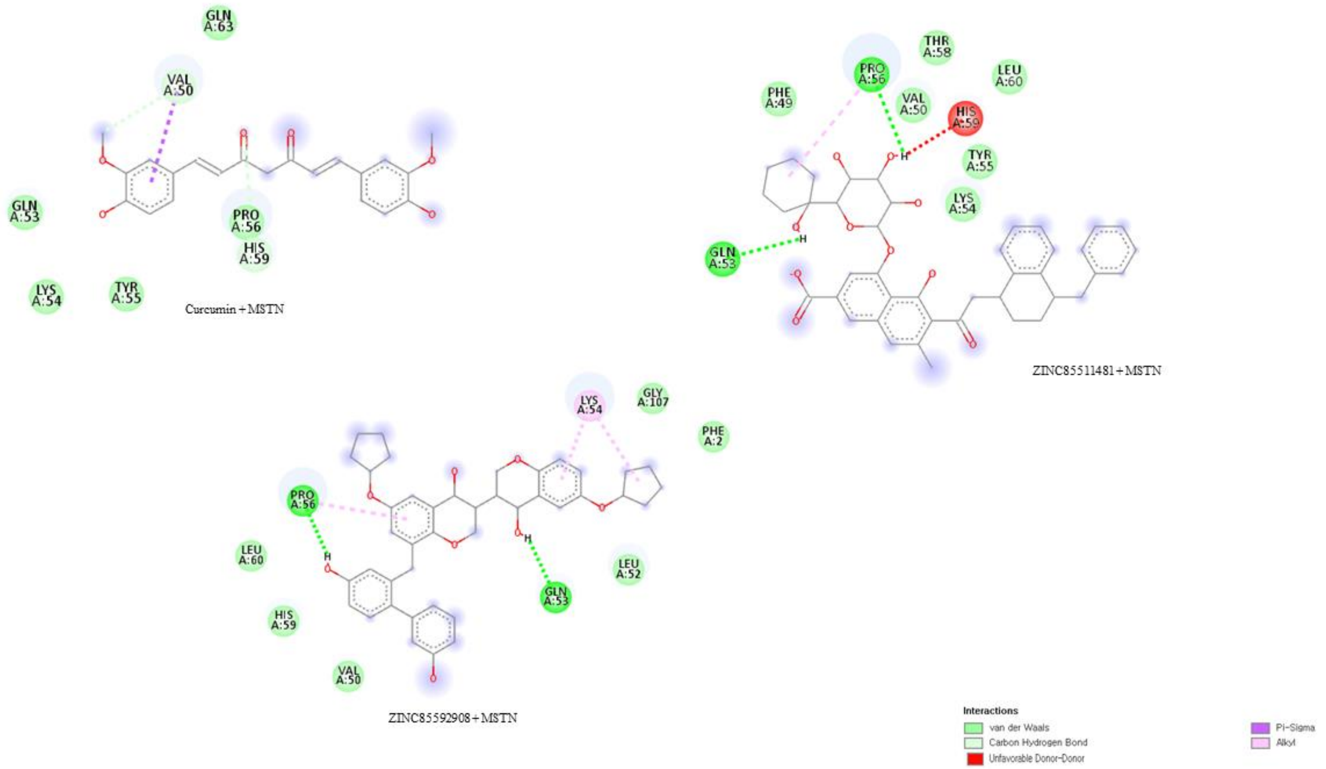

Figure S1. Leads interaction with MSTN protein.

Table S1. List of top 150 compounds showing apparent binding affinity.

S. No.	Compound ID	Affinity (kcal/mol)	S. No.	Compound ID	Affinity (kcal/mol)	S. No.	Compound ID	Affinity (kcal/mol)	S. No.	Compound ID	Affinity (kcal/mol)
1.	ZINC85531198	-13	33.	ZINC85628637	-11.6	65.	ZINC85593929	-11.2	97.	ZINC85596478	-10.9
2.	ZINC85531199	-13	34.	ZINC85531248	-11.5	66.	ZINC70454202	-11.2	98.	ZINC85569094	-10.9
3.	ZINC85531200	-12.9	35.	ZINC85531161	-11.5	67.	ZINC85530919	-11.2	99.	ZINC95913306	-10.9
4.	ZINC85531201	-12.8	36.	ZINC85531399	-11.5	68.	ZINC85592456	-11.1	100.	ZINC85625736	-10.9
5.	ZINC85531202	-12.8	37.	ZINC85949541	-11.5	69.	ZINC85542935	-11.1	101.	ZINC85593863	-10.9
6.	ZINC85531203	-12.6	38.	ZINC85542603	-11.5	70.	ZINC95911591	-11.1	102.	ZINC95910145	-10.9
7.	ZINC85531204	-12.5	39.	ZINC42802834	-11.5	71.	ZINC85844973	-11.1	103.	ZINC85593341	-10.9
8.	ZINC85531205	-12.5	40.	ZINC85595996	-11.5	72.	ZINC85543478	-11.1	104.	ZINC85568324	-10.9

9.	ZINC85531206	-12.4	41.	ZINC85531203	-11.5	73.	ZINC85531095	-11.1	105.	ZINC85593925	-10.9
10.	ZINC85531207	-12.4	42.	ZINC85542646	-11.5	74.	ZINC85542926	-11.1	106.	ZINC85531068	-10.9
11.	ZINC85531208	-12	43.	ZINC85531409	-11.4	75.	ZINC85541288	-11.1	107.	ZINC95909694	-10.9
12.	ZINC85531209	-12.4	44.	ZINC85542795	-11.4	76.	ZINC85531118	-11.1	108.	ZINC85811300	-10.9
13.	ZINC85531210	-12.2	45.	ZINC85531224	-11.4	77.	ZINC85543487	-11.1	109.	ZINC85571143_01	-10.9
14.	ZINC85531211	-12.2	46.	ZINC85542877	-11.4	78.	ZINC85569037	-11.1	110.	ZINC85811300_04	-10.9
15.	ZINC85531212	-12.2	47.	ZINC70454202_01	-11.4	79.	ZINC85592446_01	-11.1	111.	ZINC85569041_01	-10.9
16.	ZINC85531213	-12.1	48.	ZINC85531169	-11.4	80.	ZINC85531062	-11.1	112.	ZINC85531053	-10.9
17.	ZINC85531214	-12	49.	ZINC85531122	-11.4	81.	ZINC85542917	-11.1	113.	ZINC85569060	-10.9
18.	ZINC85531215	-11.9	50.	ZINC85593856	-11.4	82.	ZINC85568704	-11	114.	ZINC85569082	-10.8
19.	ZINC85531216	-11.9	51.	ZINC85542793	-11.4	83.	ZINC85531185	-11	115.	ZINC85627902_01	-10.8
20.	ZINC85531217	-11.8	52.	ZINC85592445	-11.3	84.	ZINC14680812	-11	116.	ZINC85628579_01	-10.8
21.	ZINC85991498_01	-11.8	53.	ZINC85569026_01	-11.3	85.	ZINC85628579	-11	117.	ZINC85511481	-10.8
22.	ZINC85542627	-11.8	54.	ZINC85593929_01	-11.3	86.	ZINC03780340	-11	118.	ZINC85593878_01	-10.8
23.	ZINC85592441_01	-11.7	55.	ZINC85531271	-11.3	87.	ZINC85592903	-11	119.	ZINC85542671	-10.8
24.	ZINC85531192	-11.7	56.	ZINC85531302	-11.3	88.	ZINC85593850_01	-11	120.	ZINC85542639	-10.8
25.	ZINC85531087	-11.7	57.	ZINC85531172	-11.3	89.	ZINC85542801	-11	121.	ZINC85593889_01	-10.8
26.	ZINC44086846	-11.7	58.	ZINC85568681	-11.2	90.	ZINC85593980_01	-11	122.	ZINC85593910_01	-10.8
27.	ZINC85991498	-11.7	59.	ZINC85568691	-11.2	91.	ZINC85568324_01	-11	123.	ZINC85593980	-10.8
28.	ZINC85541065	-11.7	60.	ZINC85592448	-11.2	92.	ZINC85568684	-11	124.	ZINC85531359	-10.8
29.	ZINC85596043	-11.7	61.	ZINC85542590	-11.2	93.	ZINC85531180	-11	125.	ZINC95910594	-10.8
30.	ZINC85531099	-11.6	62.	ZINC85593886	-11.2	94.	ZINC85542876	-11	126.	ZINC85593293	-10.8
31.	ZINC85542810	-11.6	63.	ZINC85628652_01	-11.2	95.	ZINC85546719	-10.9	127.	ZINC85593334	-10.8
32.	ZINC85592442_01	-11.6	64.	ZINC85531136	-11.2	96.	ZINC85628604	-10.9	128.	ZINC85532197	-10.7

**S. No.**	**Compound ID**	**Affinity (kcal/mol)**
129.	ZINC85531344	-10.8
130.	ZINC04098621	-10.8
131.	ZINC85569039_01	-10.8
132.	ZINC85592908	-10.8
133.	ZINC85543486	-10.8
134.	ZINC04098631	-10.8
135.	ZINC85542734	-10.8
136.	ZINC85592913	-10.8
137.	ZINC85542803	-10.8
138.	ZINC85593896	-10.8
139.	ZINC85627948_01	-10.8
140.	ZINC85592445_01	-10.8
141.	ZINC85569299	-10.7

142.	ZINC85592443_01	-10.7
143.	ZINC85947357_01	-10.7
144.	ZINC85593826	-10.7
145.	ZINC85596012	-10.7
146.	ZINC85532197_01	-10.7
147.	ZINC85568577	-10.7
148.	ZINC85593975_02	-10.7
149.	ZINC85593856_01	-10.7
150.	ZINC85542903	-10.7

Table S2. Percentage of secondary structure element in MSTN protein.

S. No	Compound IDs	Strand	Alpha helix	3-10 helix	Other
1.	ZINC85511481	27.5	5.5	1.8	65.1
2.	ZINC85592908	38.5	8.3	0	53.2
3.	Curcumin	39.4	5.5	0	55
